# Evidence of early subclinical atherosclerosis in women with exercise-induced hypothalamic amenorrhea

**DOI:** 10.1093/hropen/hoag055

**Published:** 2026-06-03

**Authors:** Manuel Luque-Ramírez, Sara de Lope Quiñones, Alejandra Quintero-Tobar, María Ángeles Martínez-García, María Insenser, Lía Nattero-Chávez, María Garriga, Antonio Carlos Michael Fernández, Héctor Francisco Escobar-Morreale

**Affiliations:** Diabetes, Obesity and Human Reproduction Research Group, Instituto Ramón y Cajal de Investigación Sanitaria & Centro de Investigación Biomédica en Red Diabetes y Enfermedades Metabólicas Asociadas &, Universidad de Alcalá, Madrid, Spain; Department of Endocrinology and Nutrition, Hospital Universitario Ramón y Cajal, Madrid, Spain; Diabetes, Obesity and Human Reproduction Research Group, Instituto Ramón y Cajal de Investigación Sanitaria & Centro de Investigación Biomédica en Red Diabetes y Enfermedades Metabólicas Asociadas &, Universidad de Alcalá, Madrid, Spain; Diabetes, Obesity and Human Reproduction Research Group, Instituto Ramón y Cajal de Investigación Sanitaria & Centro de Investigación Biomédica en Red Diabetes y Enfermedades Metabólicas Asociadas &, Universidad de Alcalá, Madrid, Spain; Diabetes, Obesity and Human Reproduction Research Group, Instituto Ramón y Cajal de Investigación Sanitaria & Centro de Investigación Biomédica en Red Diabetes y Enfermedades Metabólicas Asociadas &, Universidad de Alcalá, Madrid, Spain; Diabetes, Obesity and Human Reproduction Research Group, Instituto Ramón y Cajal de Investigación Sanitaria & Centro de Investigación Biomédica en Red Diabetes y Enfermedades Metabólicas Asociadas &, Universidad de Alcalá, Madrid, Spain; Diabetes, Obesity and Human Reproduction Research Group, Instituto Ramón y Cajal de Investigación Sanitaria & Centro de Investigación Biomédica en Red Diabetes y Enfermedades Metabólicas Asociadas &, Universidad de Alcalá, Madrid, Spain; Department of Endocrinology and Nutrition, Hospital Universitario Ramón y Cajal, Madrid, Spain; Department of Endocrinology and Nutrition, Hospital Universitario Ramón y Cajal, Madrid, Spain; Department of Radiodiagnostics, Hospital Universitario Ramón y Cajal, Madrid, Spain; Diabetes, Obesity and Human Reproduction Research Group, Instituto Ramón y Cajal de Investigación Sanitaria & Centro de Investigación Biomédica en Red Diabetes y Enfermedades Metabólicas Asociadas &, Universidad de Alcalá, Madrid, Spain; Department of Endocrinology and Nutrition, Hospital Universitario Ramón y Cajal, Madrid, Spain

**Keywords:** atherosclerosis, cardioautonomic function, cardiovascular risk, physical activity, functional hypothalamic amenorrhea

## Abstract

**STUDY QUESTION:**

Do women with exercise-induced functional hypothalamic amenorrhea (Ex-FHA) have an increased risk of subclinical atherosclerosis, as measured by carotid intima-media thickness (cIMT)?

**SUMMARY ANSWER:**

Women with Ex-FHA appear to show greater cIMT than non-exercising control women (nonEx-control).

**WHAT IS KNOWN ALREADY:**

Long-term cardiovascular risk is a major concern in women with functional hypothalamic amenorrhea who exercise regularly. Although hypoestrogenic women with Ex-FHA consistently exhibit markers of risk, such as endothelial dysfunction, evidence of atherosclerosis is lacking.

**STUDY DESIGN, SIZE, DURATION:**

From 2019 to 2025, we conducted a cross-sectional comparative study of five groups of premenopausal adult women (N = 50). According to our calculations, this sample size allowed us to detect a minimum difference of 0.019 mm in common cIMT between any pair of groups, setting α to 0.05 and power (1−β) to 0.80.

**PARTICIPANTS/MATERIALS, SETTING, METHODS:**

We included three groups of women with different mechanisms of ovulatory dysfunction: women with Ex-FHA, women with classic PCOS, and women with non-hyperandrogenic PCOS. Healthy exercising (Ex-control) and nonEx-control served as control groups. Smokers were excluded. All women received a comprehensive cardiometabolic phenotyping including anthropometrics, circulating sex steroids, carbohydrate metabolism and lipid profiles, office and 24-h ambulatory blood pressure monitoring, and cardioautonomic function studies. Ultrasound measurement of mean cIMT served as a marker of subclinical carotid atherosclerosis.

**MAIN RESULTS AND THE ROLE OF CHANCE:**

Women with Ex-FHA were the only subgroup of study participants showing higher cIMT values than nonEx-controls [cIMT: 0.514 ± 0.057 vs 0.411 ± 0.054 mm, respectively; mean difference: 0.103 (95% CI: 0.031; 0.175); *P *= 0.002]. This difference remained after adjusting for BMI. Regarding carbohydrate and lipid metabolism, women with Ex-FHA showed a higher insulin sensitivity index than both subgroups of women with PCOS. Individuals with Ex-FHA had higher concentrations of HDL-cholesterol, HDL-triglycerides, and large- and medium-HDL particles than both subgroups of women with PCOS, Ex-controls, and nonEx-controls. Women with Ex-FHA presented with a lower resting heart rate than participants with classic PCOS, Ex-controls, and nonEx-controls. They also showed a lower diastolic blood pressure response to standing than nonEx-controls.

**LIMITATIONS, REASONS FOR CAUTION:**

The limited sample size of our study population precluded us from identifying the main determinants of subclinical atherosclerosis in each subgroup of women. The cross-sectional observational design of this study does not allow causal inference regarding associations between independent variables and cIMT. Lastly, multiple comparisons may have led to spurious associations in some cases, despite having implemented rigorous adjustments for multiplicity whenever possible.

**WIDER IMPLICATIONS OF THE FINDINGS:**

While data on robust endpoints of women with Ex-FHA are lacking, this novel finding on subclinical carotid atherosclerosis may enhance scientific understanding of their cardiovascular risk profile. Still, larger confirmatory studies are needed with the aim of establishing cIMT as an early marker of atherosclerosis in Ex-FHA and, secondly, clarifying which intrinsic risk factors underlie this association.

**STUDY FUNDING/COMPETING INTEREST(S):**

This research was funded by Instituto de Salud Carlos III grants PI1801122 and PI2100116, and co-funded by the European Union. The authors declare not to have any conflicts of interest.

**TRIAL REGISTRATION NUMBER:**

ClinicalTrials.gov ID: NCT03841981.

WHAT DOES THIS MEAN FOR PATIENTS? In this study, we investigated whether women who lose their periods due to moderate-to-intense exercise might develop early artery disease by measuring the thickness of their carotid artery walls.

When lean, physically active women do not intake sufficient calories to meet their energy needs, they may develop a condition called functional hypothalamic amenorrhea. In this condition, the body suppresses ovarian function to save energy, which can affect important processes such as ovulation and estrogen synthesis.

We know that after menopause, lower estrogen levels can impair vascular function. This raises the question of whether reduced estrogen levels may also negatively affect cardiovascular health in women whose periods stop due to moderate-to-intense exercise.

Carotid intima-media thickness (a measure of the inner walls of the carotid arteries in the neck) is a well-known marker of early atherosclerosis and can also help predict cardiovascular events, including heart attacks and strokes.

In this study, carotid artery thickness was compared among women with hypothalamic amenorrhea due to doing moderate-to-intense exercise and three other groups: women with polycystic ovary syndrome (a condition which may also be associated with increased carotid thickness), women who exercise and have regular periods, and healthy women with regular periods who do not exercise.

The researchers concluded that exercising women with functional hypothalamic amenorrhea had thicker carotid artery walls than sedentary control women. This novel finding may provide evidence that a chronic negative energy balance could carry cardiovascular risks even in non-overweight women who perform moderate-to-intense exercise.

## Introduction

Aside from bone health, long-term cardiovascular (CV) risk is a major concern in exercising women who suffer from functional hypothalamic amenorrhea (Ex-FHA) and, consequently, from hypoestrogenism (Shufelt *et al.*, 2023; Tegg *et al.*, 2024). CV disease clearly shows sexual dimorphism, as CV events are more prevalent in men than in women of reproductive age (Kappert *et al.*, 2012; Martin *et al.*, 2025). However, after menopause, the lifetime risk of CV disease between women and men is equated (Leening *et al.*, 2014; Martin *et al.*, 2025). Further supporting the detrimental role of hypoestrogenism in CV disease in women, premature ovarian insufficiency—formerly termed premature menopause—and early-onset menopause also increase the risk of atherosclerotic CV disease (Muka *et al.*, 2016; Honigberg *et al.*, 2019). Similarly, a shorter duration of reproductive lifespan is associated with a higher risk of CV disease (Ley *et al.*, 2017).

Regular moderate-to-vigorous exercise is promoted by health providers because of its positive effect on decreasing CV risk and supporting secondary prevention (Pelliccia *et al.*, 2021; Broderick *et al.*, 2025). It also shares some CV benefits with endogenous estrogens (Moreira *et al.*, 2020; Laird *et al.*, 2025). Moreover, there is strong evidence that sedentary behavior is a well-known CV risk factor (Martin *et al.*, 2025). However, lifelong endurance sport participation may be counterintuitively associated with more coronary plaques, including more risk-prone non-calcified coronary plaques in proximal segments, according to evidence comparing lifelong endurance athletes with similarly fit and healthy individuals with a low CV risk profile (De Bosscher *et al.*, 2023). The downregulation of gonadal axis in women with Ex-FHA might even boost this paradoxical effect of exercise on vascular atherosclerosis, since estrogen deficiency in these women is accompanied by a lower flow-mediated vascular dilation suggestive of endothelial dysfunction (Tegg *et al.*, 2024).

Hence, whether women with Ex-FHA present with signs of subclinical atherosclerosis becomes a relevant research question. To provide new insights into this issue, we conducted a cross-sectional comparative study with the following specific aims:

- To measure the carotid intima-media thickness (cIMT)—a surrogate marker of subclinical atherosclerosis—in women with Ex-FHA and compare it with individuals with PCOS, which is also associated with increased cIMT (Luque-Ramírez *et al.*, 2007), and with control groups of non-exercising (nonEx-controls) and exercising (Ex-controls) women presenting regular menses.
- To define the role of Ex-FHA on carbohydrate metabolism, lipid profile, blood pressure (BP) recordings, and cardioautonomic function compared with the groups of women described above.
- To identify the associations between cIMT and these CV risk factors.

## Materials and methods

### Ethics

All experiments were performed in accordance with relevant guidelines and regulations, including the Declaration of Helsinki and the Spanish law 3/2018 on the Protection of Personal Data in accordance with the European Union Regulation (EU 2016/679) on Data Protection. All patients and controls provided informed consent allowing them to participate in this study. The research protocol was approved by the local Ethics Committee from Hospital Universitario Ramón y Cajal (Date of approval: 17 December 2018; Reference number: 2018/0038).

### Study design

This report is part of a broader cross-sectional study designed to address the role of the amount, distribution, and dysfunction of body fat as determinants of gonadal dysfunction in women (ClinicalTrials.gov ID: NCT03841981). With this aim, the current study included three groups of women with ovulatory dysfunction and diverse pathogenic background (women with Ex-FHA, classic PCOS, and non-hyperandrogenic PCOS), and two groups of control women (Ex-controls and nonEx-controls).

### Subjects

**Figure 1.**
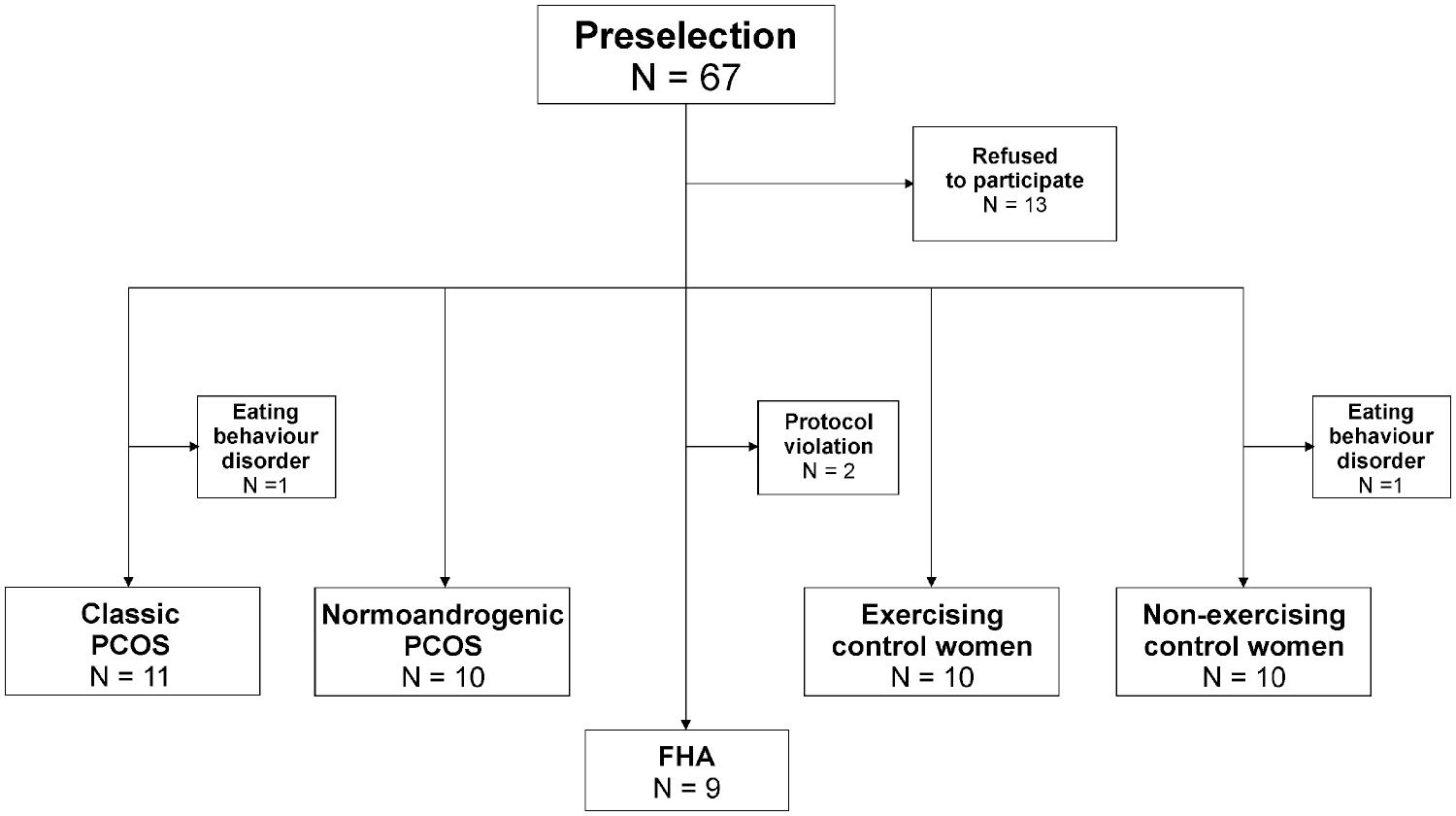
**Flow of study participants’ recruitment**. FHA, functional hypothalamic amenorrhea.

From 2019 to 2025, we prospectively recruited premenopausal women aged 18–45 years attending the Reproductive Endocrinology clinic of an academic hospital in Madrid, Spain (Fig. 1).

Ex-FHA was diagnosed in amenorrheic individuals (no menses for ≥3 months) who self-reported ≥3 h/week of moderate-to-vigorous intensity physical activity (Piercy *et al.*, 2018), and who had: (i) FSH and LH concentrations below 10 IU/l; (ii) circulating estradiol (E_2_) below 92 pM (25 pg/ml); and (iii) lack of bleeding after an oral progesterone withdrawal challenge.

These women reported a median duration of amenorrhea of 22 months, with 25th–75th percentile values of 13 and 39 months, respectively. Other causes of secondary amenorrhea were ruled out, including hyperprolactinemia, thyroid dysfunction, pregnancy, PCOS, and non-classic congenital adrenal hyperplasia.

A diagnosis of classic PCOS required the presence of at least clinical and/or biochemical androgen excess, and ovulatory dysfunction (OD), independently of the presence of polycystic ovarian morphology (PCOM). Women with normoandrogenic PCOS were defined as those presenting with OD and PCOM, in the absence of clinical signs of hyperandrogenism and hyperandrogenemia. Both definitions fulfilled the accepted current consensus for PCOS diagnosis (Teede *et al.*, 2023). Individuals with PCOS were diagnosed by state-of-the-art methodology including LC-MS/MS assays for biochemical hyperandrogenism and assessment of PCOM by sonography and/or anti-Müllerian hormone immunoassay (Luque-Ramírez *et al.*, 2025). Biochemical hyperandrogenism and upper limit of normality, respectively, were established by the presence of values > 95th percentile of a sample of control women (Luque-Ramírez *et al.*, 2025). OD (corresponding to World Health Organization group II ovulation disorders) was defined by the presence of more than six cycles longer than 36 days in the previous year, absence of menstruation for three consecutive months, or by two consecutive luteal phase progesterone concentrations below 17 nM in women presenting with regular menses. PCOM was evaluated by gynecologists according to routine clinical practice based on the current evidence-based definition (Teede *et al.*, 2023). We systematically excluded women with other etiologies of hyperandrogenism or OD (Escobar-Morreale, 2018; Teede *et al.*, 2023).

To serve as Ex-control and nonEx-control women, we recruited premenopausal volunteers. Exercise requirements were the same as those requested for Ex-FHA patients. Both groups of controls presented no signs of androgen excess in physical examination, reported regular menses, and were assessed using the same methods applied to other study subjects.

Patients and controls had neither a history of oophorectomy or hysterectomy, nor had they received treatment with hormonal contraceptives, hormone replacement therapy, antiandrogens, or insulin sensitizers for at least 6 months before recruitment. Before that date, seven participants with Ex-FHA reported earlier treatment with combined oral contraceptives, with a median duration of treatment of 4.5 years and ranging from 0.6 to 11 years, and one woman had received hormone replacement therapy for 6 months.

All study participants had a BMI > 18 kg/m^2^, had no history of eating disorders or smoking, and were not previously diagnosed with prediabetes/diabetes, hypertension, or dyslipidemia.

### Procedures

A trained investigator (M.L.-R.) was responsible for the clinical, anthropometric, and physical evaluations including hirsutism score, BMI, and waist circumference. The percentages of body fat and lean mass with respect to total body weight and phase angle were determined using a bioelectrical impedance (BIE) analyzer (Medeia^®^ VitalScan Monitor System device; VitalScan, Santa Barbara, CA, USA).

### Sampling

Serum and plasma samples were obtained between Days 3 and 9 of spontaneous menstrual bleeding or after excluding pregnancy in amenorrheic patients. After a 3-day 300-g carbohydrate diet and 12-h overnight fasting, we obtained blood samples for a sex steroid profile consisting of serum total testosterone (T), sex hormone-binding globulin (SHBG) and calculated free T, androstenedione (A4), dehydroepiandrosterone-sulfate (DHEA-S), estrone (E_1_) and E_2_, and a complete lipid profile. Serum 17-hydroxyprogesterone, prolactin, LH, FSH, and thyroid-stimulating hormone were also measured. Then, a 75-g oral glucose tolerance test (OGTT) was performed, and samples were obtained for measurement of plasma insulin and glucose at 0, 30, 60, 90, and 120 min. Samples were immediately centrifuged, and serum and plasma were aliquoted, coded, and frozen at −80°C until thawed for analysis.

Homeostasis model assessment of insulin resistance (HOMA-IR) was calculated from fasting (0 min) plasma glucose and insulin. The composite insulin sensitivity index (Matsuda index) was calculated from the circulating glucose and insulin concentrations during the OGTT as an estimation of whole-body insulin sensitivity (Matsuda and DeFronzo, 1999). The areas under the curve (AUC) for glucose and insulin during the OGTT were determined using the trapezoidal rule.

### Assays

The laboratory personnel who ran all the assays were blinded to the women’s features and group allocation. Stored aliquots of serum samples were assayed for total T, A4, E_1_, and E_2_ by LC–MS/MS at the Laboratory of Hormonology of the University of Ghent, Belgium, using an AB Sciex 6500 triple-quadrupole mass spectrometer (AB Sciex, Toronto, Canada). The lower limit of quantification (LLOQ) was 0.04 nM (1.2 ng/dl) for total T and the inter-assay CV was 8.3% at 1.27 nM (36.6 ng/dl) and 3.1% at 10.7 nM (308.4 ng/dl). Serum LLOQ was <0.17 nM (0.05 ng/ml) for A4 and the inter-assay CV was <7.1%. E_2_ had a LLOQ of 1.10 pM (0.3 pg/ml), and inter-assay CV of 6.73% at 14.2 nM (3854 pg/ml), 10% at 0.16 nM (44 pg/ml), and 6.59% at 1.20 nM (326 pg/ml). Corresponding figures for E_1_ were: LLOQ of 1.11 pM (0.3 pg/ml); inter-assay CV of 10.5% at 0.10 nM (27 pg/ml), 10.1% at 12.6 nM (3414 pg/ml), and 9.47% at 0.97 nM (261 pg/ml). We calculated free T concentrations from total T measured by routine immunoassays and LC-MS/MS assays, SHBG concentrations, and albumin (Vermeulen *et al.*, 1999). SHBG was measured using an automated chemiluminescent immunoassay (IMMULITE 2000, Siemens Healthcare Sector, Erlangen, Germany) with a LLOQ of 0.02 nM and mean intra-assay and inter-assay CVs < 10%.

Circulating apolipoprotein (Apo) AI, Apo B100, and lipoprotein (a) levels were determined by kinetic immunonephelometry (Dade Behring, Deerfield, IL, USA). The lipid composition [cholesterol (C) and triglycerides (TG)], mean size, and number of particles (P) of the three main lipoprotein [very low-density lipoprotein (VLDL), low-density lipoprotein (LDL), and high-density lipoprotein (HDL)] were assayed using the Liposcale^®^ test (Biosfer Teslab, Reus, Tarragona, Spain), yielding nine lipoprotein subtypes (i.e. large, medium, and small VLDL, LDL, and HDL). The lipid composition of intermediate-density lipoprotein subfraction (IDL) was also assessed (Mallol *et al.*, 2015).

### Blood pressure measurements

Blood pressure (BP) and heart rate (HR) were measured three times, 1 min apart, using a calibrated automatic digital sphygmomanometer (Welch Allyn Spot Vital Signs 4200B, Welch Allyn, Skaneateles Falls, NY, USA) in the non-dominant arm, with a proper cuff; women were seated for at least 5 min before taking any reading. The average of the three measurements was used as an estimation of office systolic and diastolic BP, and HR at resting.

Twenty-four-hour BP recordings were obtained using an A&D TM2430EX oscillometric device (A&D Company, Ltd., Tokyo, Japan). The cuff (12 × 22 cm for lean patients, and 14 × 30 cm for overweight or obese patients) was placed on the non-dominant arm. The period from 07:00 to 23:00 was considered daytime, and from 23:00 until 07:00 the next day was considered nighttime, reflecting the usual sleeping habits in Spain. Systolic, diastolic, and mean BP as well as HR were measured every 20 min during daytime and every 30 min during nighttime.

### Cardiovascular autonomic function assessment

Cardioautonomic function (parasympathetic innervation) was evaluated using gold standard methods (Ewing *et al.*, 1985; Pop-Busui *et al.*, 2017): (i) the standardized cardiac autonomic reflex tests described by Ewing *et al.* (Ewing *et al.*, 1985) in 1970, and (ii) power spectral HR variability by analyzing beat-to-beat intervals from short-duration electrocardiogram (EKG) recordings.

Between 7:00 and 9:00 a.m., and after resting in supine for at least 10 min in a room with stable temperature, we assessed HR variability using a Medeia VitalScan^®^ Monitor System device (VitalScan). Participants were instructed to avoid food and caffeine for the 12 h preceding these procedures. The HR response during deep breathing was established by calculating the ratio of the maximum and minimum HR during six cycles of paced deep breathing [expiratory/inspiratory (E/I) ratio]. Second, HR response to Valsalva’s maneuver was assessed by calculating the ratio of the longest R–R interval after the maneuver to the shortest interval during or shortly after the maneuver. HR response to standing (30:15 ratio) was calculated as the ratio of the longest R-R interval (found at approximately beat 30) to the shortest interval (found at approximately beat 15) after standing up. We also obtained power spectral HR data by analyzing the time series of beat-to-beat intervals from short-duration EKG recordings (10 min) using specialized frequency-domain software VitalScan Medeia^®^ (VitalScan). This analysis uses the Fourier method, which transforms R–R intervals into wavelets with two basic components: low frequency (LF) 0.04–0.15 Hz and high frequency (HF) 0.15–0.40 Hz. LF activity represents the combined effects of sympathetic and parasympathetic influence, whereas HF represents parasympathetic activity.

Finally, we assessed changes in BP and HR by comparing the values observed 5 min after active standing with those recorded while resting in supine. During a normal physiologic response to standing, there is a rapid and brief decrease in BP, along with a rapid increase in HR to compensate for the reduced BP and cardiac output. An augmented vagal basal tone and/or diminished sympathetic response may signal an abnormal response to standing.

### Carotid intima-media thickness assessment

Imaging was conducted using a high-resolution 7–15 MHz phased-array transducer (EPIQ 5, Philips Healthcare, Bothell, WA, USA) by the same trained operator (A.C.M.F.) in all study participants. This operator was blinded to the women’s features and their group allocation. Under controlled light and temperature conditions, studies were performed by positioning the women in a supine position with a 35-degree incline of the head and torso, and a 45 degrees right-turn or left-turn, respectively, of the head. The left and right common carotid arteries were explored in B-mode in longitudinal and transversal planes, to rule out the presence of plaque that might interfere with cIMT measurements. The posterior carotid wall at 1 cm of the common carotid bulb was imaged and cIMT was estimated by visual assessment (average of five manual measurements) of the distance between the lumen/intima and intima/adventitia interphases in longitudinal frames acquired during arterial diastole.

### Sample size

We used data from previous local research comparing the cIMT of patients with PCOS and non-hyperandrogenic women (Luque-Ramírez *et al.*, 2007) using the online sample size and power calculator version 8.0 developed by the Institut Municipal d’Investigació Mèdica in Barcelona, Spain (https://www.datarus.eu/aplicaciones/granmo/). Assuming a common SD of 0.011 (Luque-Ramírez *et al.*, 2007), eight subjects would be necessary in each subgroup to recognize a minimum difference above 0.019 mm between any pair of groups, setting α to 0.05 and power (1−β) to 0.80.

### Statistical analysis

Data are shown as mean ± SD or 95% CIs, and counts (percentage). For continuous variables, their normal distribution was assessed by the Kolmogorov–Smirnov test, and logarithmic transformation was applied to ensure normality if needed. Continuous variables were compared by Kruskal–Wallis’s *H* tests, one-way ANOVA, Welch’s ANOVA, or univariate general linear models as a function of the distribution, homogeneity of variances and adjustment by covariates. Pair-wise mean differences between exercising women with Ex-FHA and other study subgroups were analyzed by *post hoc* Tukey, Games-Howell, or Bonferroni methods. Pearson’s or Spearman’s correlation analyses served to describe the association between CV risk factors and cIMT considering all study participants as a whole. We performed statistical analyses using IBM^®^ SPSS^®^ Statistics 23 (IBM España S.A., Madrid, Spain). A *P*-value < 0.05 was considered statistically significant.

## Results

The anthopometrics and sex steroid profiles of the study subgroups are shown in Table 1. Of note, women with Ex-FHA had lower BMI and fat mass percentages than both subgroups of patients with PCOS and nonEx-controls, but similar to those of Ex-controls. As expected, LH concentrations and LH-to-FSH ratios were lower in amenorrheic patients with Ex-FHA compared with all the other study subgroups. Lastly, women with Ex-FHA showed lower E_2_ and E_1_ concentrations than patients with classic PCOS, nonEx-controls, and Ex-control women.

**Table 1.** Anthropometrics and sex steroids profiles of women with exercise-induced functional hypothalamic amenorrhea compared to individuals with polycystic ovary syndrome and control participants.

	Participants with ovulatory dysfunction			Control participants		F/χ^2^	*P*
	Ex-FHA	Normoandrogenic PCOS	Classic PCOS	Ex-controls	nonEx-controls		
	(N = 9)	(N = 10)	(N = 11)	(N = 10)	(N = 10)		
Age, years	30.0 ± 3.3	27.2 ± 5.9	22.0 ± 3.5*	27.4 ± 7.3	24.5 ± 4.4	3.6	0.012
Anthropometry							
BMI, kg/m^2^^a^	20.0 ± 1.1	27.9 ± 5.3*	27.9 ± 7.5*	21.8 ± 2.4	22.9 ± 1.9*	11.2	<0.001
Waist circumference, cm^a^	69 ± 4	90 ± 14*	86 ± 18	73 ± 7	75 ± 7	6.7	0.001
Waist-to-hip ratio	0.81 ± 0.08	0.82 ± 0.07	0.80 ± 0.09	0.76 ± 0.03	0.76 ± 0.05	8.4	0.080
Biolelectrical impedance analysis^†^							
Fat mass, %^a^^,^^c^^,^^d^	19.7 ± 3.7	33.6 ± 6.2*	31.7 ± 6.4*	21.9 ± 3.5	28.0 ± 2.6*	32.7	<0.001
Lean mass, %	72.8 ± 23.5	61.5 ± 13.8	58.4 ± 20.7	72.3 ± 18.7	55.7 ± 25.6	1.4	0.253
Phase angle 50 KHz	7.6 ± 2.1	6.7 ± 0.6	6.3 ± 0.7	7.7 ± 1.6	7.9 ± 2.5	4.6	0.331
Sex steroids and gonadal axis							
LH, UI/l	1.6 ± 1.0	4.9 ± 1.6*	6.8 ± 4.0*	4.0 ± 1.7*	4.2 ± 1.3*	11.5	<0.001
FSH, UI/l	4.8 ± 1.8	5.7 ± 0.7	5.2 ± 2.2	6.5 ± 1.8	6.0 ± 1.5	5.9	0.208
LH-to-FSH ratio	0.31 ± 0.14	0.86 ± 0.28*	1.48 ± 0.92*	0.61 ± 0.21*	0.71 ± 0.21*	12.7	<0.001
Estradiol, pM	44 ± 37	132 ± 99	213 ± 169*	143 ± 81*	154 ± 96*	19.2	0.001
Estrone, pM^c^	78 ± 22	178 ± 133	237 ± 93*	129 ± 41*	166 ± 70*	8.8	<0.001
Total testosterone, nM^e^^,^^f^	0.59 ± 0.16	0.83 ± 0.31	1.35 ± 0.35*	0.98 ± 0.27	0.90 ± 0.35	8.4	<0.001
Sex hormone binding globulin, nM^e^	55 ± 10	53 ± 35	38 ± 20	70 ± 35	61 ± 19	3.0	0.040
Calculated free testosterone, pM^c^^,^^e^^,^^f^	8 ± 2	12 ± 5	23 ± 7*	12 ± 5	11 ± 5	22.4	<0.001
Androstenedione, nM^c^^,^^e^^,^^f^	2.9 ± 0.8	4.2 ± 1.2	6.8 ± 2.2*	4.6 ± 1.3	4.5 ± 2.2	7.5	<0.001
Dehydroepiandrosterone-sulphate, µM	4.1 ± 1.7	5.1 ± 1.7	8.5 ± 2.8*	5.2 ± 1.7	5.6 ± 2.3	3.4	0.017
Anti-Müllerian hormone, pM^b^^,^^c^^,^^e^	21 ± 15	40 ± 17	55 ± 33	21 ± 10	17 ± 7	6.4	0.002

Data are means ± SD. Variables were compared among groups by Kruskall–Wallis’s *H*, univariate ANOVA, Welch-ANOVA, or univariate-GLM analysis adjusted by age if necessary, and followed by Tukey’s, Games-Howell’s, or Bonferroni’s *post hoc* analysis, as appropriate.

Ex-controls, healthy exercising control women with regular menses; Ex-FHA, women with exercise-induced functional hypothalamic amenorrhea; nonEx-controls, non-exercising control women with regular menses.

Pair-wise comparisons between women with Ex-FHA and other study subgroups:.

* Participants showing statistically significant differences (*P *< 0.05) compared with women with Ex-FHA.

a Statistically significant differences (*P *< 0.05) between Ex-control women and normoandrogenic PCOS.

b Statistically significant differences (*P *< 0.05) between nonEx-control women and normoandrogenic PCOS.

c Statistically significant differences (*P *< 0.05) between Ex-control women and classic PCOS.

d Statistically significant differences (*P *< 0.05) between Ex-control and nonEx-control women.

e Statistically significant differences (P <0.05) between nonEx-control women and classic PCOS.

f Statistically significant differences (P <0.05) between normoandrogenic and classic PCOS.

† Data from one non-exercising control woman was not available for technical reasons.

Regarding carbohydrate and lipid metabolism (Table 2), women with Ex-FHA showed lower basal insulin and HOMA-IR index than participants with classic PCOS, and a higher insulin sensitivity index than both subgroups of women with PCOS. Likewise, individuals with Ex-FHA showed higher concentrations of HDL-cholesterol, HDL-triglycerides, and large- and medium-HDL particles than both subgroups of women with PCOS, Ex-controls, and nonEx-control women. Accordingly, their Apo A-I concentrations were also higher than those of both groups of women with PCOS. Finally, medium LDL particles and LDL size were higher in women with Ex-FHA compared with those with classic PCOS, whereas small-VLDL particles were lower in the former compared with the latter.

**Table 2.** Carbohydrate metabolism and advanced lipid profiles of women with exercise-induced functional hypothalamic amenorrhea compared to individuals with polycystic ovary syndrome and control participants.

	Participants with ovulatory dysfunction			Control participants		F/χ^2^	*P*
	Ex-FHA	Normoandrogenic PCOS	Classic PCOS	Ex-controls	nonEx-controls		
	(N = 9)	(N = 10)	(N = 11)	(N = 10)	(N = 10)		
Carbohydrate metabolism							
Basal plasma glucose, mM	4.6 ± 0.6	4.8 ± 0.4	4.6 ± 0.3	4.6 ± 0.3	4.7 ± 0.5	0.5	0.711
Basal plasma insulin, pM^c^	25 ± 8	50 ± 27	76 ± 49*	24 ± 9	39 ± 14	6.0	0.002
Homeostasis model assessment of insulin resistance^c^	0.8 ± 0.4	1.6 ± 0.9	2.2 ± 1.4*	0.7 ± 0.3	1.2 ± 4.5	6.1	0.002
AUC-OGGT_glucose_, mM*120 min^-1^	97 ± 123	208 ± 114	224 ± 95	160 ± 130	194 ± 161	1.5	0.212
AUC-OGGT_insulin,_ nM*120 min^-1^	21.80 ± 13.25	47.28 ± 34.07	50.77 ± 36.62	23.18 ± 9.03	23.61 ± 9.88	2.8	0.039
Insulin sensitivity index^c^	13.2 ± 4.7	6.9 ± 4.5*	4.9 ± 2.6*	12.1 ± 5.6	8.8 ± 3.4	6.9	<0.001
Lipid profile							
Total cholesterol, mM	5.8 ± 0.6	5.4 ± 0.5	5.0 ± 0.4*	5.3 ± 0.6	5.3 ± 0.4	9.9	0.042
High-density lipoprotein-cholesterol, mM^c^^,^^e^	2.0 ± 0.2	1.6 ± 0.2*	1.4 ± 0.2*	1.7 ± 0.1*	1.7 ± 0.1*	9.5	<0.001
Intermediate-density lipoprotein-cholesterol, mM	0.20 ± 0.09	0.16 ± 0.04	0.13 ± 0.04	0.17 ± 0.06	0.14 ± 0.05	2.2	0.079
Low-density lipoprotein-cholesterol, mM	3.4 ± 0.5	3.4 ± 0.5	3.1 ± 0.4	3.2 ± 0.5	3.3 ± 0.3	4.0	0.409
Very low-density lipoprotein-cholesterol, mM^e^	0.24 ± 0.11	0.25 ± 0.09	0.40 ± 0.13	0.27 ± 0.07	0.24 ± 0.08	3.1	0.023
Total triglycerides, mM	0.92 ± 0.21	0.81 ± 0.15	1.01 ± 0.27	0.84 ± 0.14	0.76 ± 0.14	2.1	0.122
High-density lipoprotein-triglycerides, mM	0.19 ± 0.03	0.13 ± 0.02*	0.14 ± 0.02*	0.16 ± 0.03*	0.14 ± 0.02*	10.9	<0.001
Intermediate-density lipoprotein-triglycerides, mM	0.10 ± 0.03	0.08 ± 0.01	0.07 ± 0.01	0.09 ± 0.02	0.08 ± 0.02	1.8	0.171
Low-density lipoprotein-triglycerides, mM^e^^,^^f^	0.20 ± 0.05	0.15 ± 0.03	0.12 ± 0.03*	0.16 ± 0.04	0.15 ± 0.01	6.0	0.002
Very low-density lipoprotein-triglycerides, mM	0.43 ± 0.16	0.44 ± 0.14	0.67 ± 0.26	0.43 ± 0.07	0.39 ± 0.11	2.5	0.074
Particle subclasses							
Large very low-density lipoprotein particles, nM^e^	0.74 ± 0.30	0.84 ± 0.27	1.27 ± 0.41	0.83 ± 0.12	0.73 ± 0.22	3.2	0.023
Medium very low-density lipoprotein particles, nM	2.32 ± 1.28	2.14 ± 1.18	3.58 ± 1.82	2.21 ± 0.82	2.00 ± 0.93	2.1	0.095
Small very low-density lipoprotein particles, nM^c^^,^^e^^,^^f^	24.8 ± 9.3	25.9 ± 8.0	39.4 ± 14.7*	25.7 ± 4.3	23.1 ± 6.3	5.3	0.001
Large low-density lipoprotein particles, nM	217.8 ± 29.5	205.0 ± 25.0	185.9 ± 22.5	205.9 ± 25.0	205.1 ± 18.1	2.2	0.085
Medium low-density lipoprotein particles, nM	462.8 ± 110.7	415.5 ± 90.7	305.4 ± 79.2*	393.1 ± 112.3	395.6 ± 53.8	4.0	0.007
Small low-density lipoprotein particles, nM^c^	590.8 ± 50.6	647.1 ± 75.6	661.7 ± 79.4	576.9 ± 56.1	604.9 ± 68.0	3.0	0.028
Large high-density lipoprotein particles, µM	0.38 ± 0.06	0.29 ± 0.04*	0.28 ± 0.04*	0.30 ± 0.04*	0.28 ± 0.02*	15.3	0.004
Medium high-density lipoprotein particles, µM	15.8 ± 2.2	11.2 ± 2.3*	9.9 ± 1.1*	12.4 ± 1.1*	12.3 ± 1.5*	11.6	<0.001
Small high-density lipoprotein particles, µM	19.9 ± 2.3	17.7 ± 3.0	16.5 ± 3.0	18.0 ± 1.5	18.3 ± 2.5	2.3	0.074
Particle size							
Very low-density lipoprotein, nm	41.95 ± 0.22	41.91 ± 0.25	42.00 ± 0.23	41.96 ± 0.26	41.94 ± 0.26	0.2	0.949
Low-density lipoprotein, nm^c^^,^^e^^,^^f^	21.42 ± 0.21	21.21 ± 0.14	20.97 ± 0.25*	21.35 ± 0.17	21.29 ± 0.14	8.6	<0.001
High-density lipoprotein, nm	8.37 ± 0.06	8.30 ± 0.04	8.29 ± 0.06	8.32 ± 0.04	8.32 ± 0.08	9.6	0.047
Apolipoproteins							
Apolipoprotein A-I, g/l^e^	2.04 ± 0.33	1.63 ± 0.30*	1.42 ± 0.20*	1.72 ± 0.15	1.89 ± 0.46	6.1	0.001
Apolipoprotein B100, g/l	0.83 ± 0.17	0.89 ± 0.17	0.77 ± 0.10	0.76 ± 0.18	0.89 ± 0.34	1.1	0.385
Lipoprotein (a), mg/l^e^	3.4 ± 3.4	3.1 ± 2.4	0.8 ± 0.7	1.8 ± 2.7	4.1 ± 3.6	9.9	0.043

Data are means ± SD. Variables were compared among groups by Kruskall–Wallis’s *H*, univariate ANOVA, Welch-ANOVA, or univariate-GLM analysis adjusted by age if necessary, and followed by Tukey’s, Games-Howell’s, or Bonferroni’s *post hoc* analysis, as appropriate.

AUC, area under the curve; Ex-controls, healthy exercising control women with regular menses; Ex-FHA, women with exercise-induced functional hypothalamic amenorrhea; nonEx-controls, non-exercising control women with regular menses; OGTT, oral glucose tolerance test.

Pair-wise comparisons between women with Ex-FHA and other study subgroups.

* Participants showing statistically significant differences (*P *< 0.05) compared with women with Ex-FHA.

a Statistically significant differences (*P *< 0.05) between Ex-control women and normoandrogenic PCOS.

b Statistically significant differences (*P *< 0.05) between nonEx-control women and normoandrogenic PCOS.

c Statistically significant differences (*P *< 0.05) between Ex-control women and classic PCOS.

d Statistically significant differences (*P *< 0.05) between Ex-control and nonEx-control women.

e Statistically significant differences (*P *< 0.05) between nonEx-control women and classic PCOS.

f Statistically significant differences (*P *< 0.05) between normoandrogenic and classic PCOS.

Women with Ex-FHA had a lower resting HR than participants with classic PCOS, Ex-control, and nonEx-control women (Table 3). They also showed a lower diastolic BP response to standing than nonEx-control women.

**Table 3.** Blood pressure recordings and cardioautonomic parameters of women with exercise-induced functional hypothalamic amenorrhea compared to individuals with polycystic ovary syndrome and control participants.

	Participants with ovulatory dysfunction			Control participants		F/χ^2^	*P*
	Ex-FHA	Normoandrogenic PCOS	Classic PCOS	Ex-controls	nonEx-controls		
Blood pressure recordings	(N = 9)	(N = 10)	(N = 11)	(N = 10)	(N = 10)		
Office							
Systolic blood pressure, mmHg	104 ± 7	110 ± 10	111 ± 6	108 ± 5	114 ± 9	2.2	0.086
Diastolic blood pressure, mmHg	69 ± 7	70 ± 8	67 ± 10	69 ± 4	71 ± 5	0.4	0.784
Heart rate, bpm	58 ± 9	71 ± 10	70 ± 8*	64 ± 9*	75 ± 11*	5.1	0.002
Ambulatory blood pressure monitoring							
Daytime							
Mean systolic blood pressure, mmHg	121 ± 11	116 ± 10	121 ± 13	121 ± 9	119 ± 10	0.5	0.748
Mean SD, mmHg	30 ± 8	22 ± 6	21 ± 9	23 ± 7	23 ± 6	1.7	0.176
Systolic blood pressure load, %	25 ± 14	16 ± 11	23 ± 19	17 ± 11	22 ± 12	0.6	0.701
Mean diastolic blood pressure, mmHg	75 ± 6	71 ± 7	74 ± 8	74 ± 4	72 ± 5	1.7	0.795
Mean SD, mmHg	25 ± 5	18 ± 7	17 ± 10	20 ± 6	19 ± 7	1.6	0.199
Diastolic blood pressure load, %	18 ± 9	13 ± 12	21 ± 26	14 ± 8	14 ± 10	0.5	0.759
Mean heart rate, bpm	69 ± 8	77 ± 10	78 ± 9	69 ± 9	78 ± 8	3.2	0.020
Mean SD, bpm	21 ± 4	13 ± 3*	16 ± 7	13 ± 3*	15 ± 3*	22.3	<0.001
Nighttime							
Mean systolic blood pressure, mmHg	100 ± 8	101 ± 12	101 ± 12	100 ± 10	96 ± 8	0.9	0.499
Mean SD, mmHg	12 ± 5	12 ± 5	13 ± 6	10 ± 4	12 ± 6	0.3	0.847
Systolic blood pressure load, %	9 ± 8	9 ± 10	13 ± 20	9 ± 18	7 ± 10	0.9	0.503
Mean diastolic blood pressure, mmHg	57 ± 3	58 ± 7	59 ± 6	59 ± 11	56 ± 6	0.5	0.761
Mean SD, mmHg	7 ± 2	10 ± 6	11 ± 6	12 ± 8	10 ± 6	0.9	0.503
Diastolic blood pressure load, %	2 ± 4	10 ± 12	8 ± 9	13 ± 24	9 ± 10	0.8	0.545
Mean heart rate, bpm	58 ± 10	64 ± 9	65 ± 10	54 ± 10	62 ± 8	2.3	0.067
Mean SD, bpm	7 ± 5	9 ± 4	7 ± 4	10 ± 3	8 ± 3	0.9	0.480
24 h							
Mean systolic blood pressure, mmHg	116 ± 9	111 ± 8	116 ± 13	115 ± 8	114 ± 9	0.3	0.851
Mean SD, mmHg	28 ± 8	22 ± 6	22 ± 8	23 ± 7	23 ± 6	1.4	0.239
Systolic blood pressure load, %	21 ± 10	18 ± 19	14 ± 8	14 ± 9	17 ± 11	1.0	0.404
Mean diastolic blood pressure, mmHg	70 ± 6	67 ± 5	70 ± 7	70 ± 4	69 ± 5	1.9	0.755
Mean SD, mmHg	23 ± 5	18 ± 6	19 ± 9	20 ± 6	19 ± 6	0.9	0.497
Diastolic blood pressure load, %	14 ± 7	13 ± 11	15 ± 13	13 ± 6	13 ± 9	0.3	0.877
Mean heart rate, bpm	65 ± 7	74 ± 9	74 ± 9	66 ± 8	75 ± 8	11.2	0.025
Mean SD, bpm	20 ± 3	14 ± 3*	25 ± 29	13 ± 3*	16 ± 2*	22.2	0.001
Nocturnal decrease in mean blood pressure, mmHg	−21 ± 7	−15 ± 12	−18 ± 8	−19 ± 12	−21 ± 6	0.6	0.671
Cardiac autonomic function tests							
Expiratory-Inspiratory ratio	1.47 ± 0.17	1.38 ± 0.15	1.45 ± 0.15	1.52 ± 0.19	1.48 ± 0.16	1.1	0.383
Valsalva test	1.38 ± 0.12	1.44 ± 0.19	1.43 ± 0.29	1.52 ± 0.20	1.42 ± 0.31	0.5	0.742
30:15 ratio	1.31 ± 0.20	1.37 ± 0.15	1.38 ± 0.19	1.40 ± 0.16	1.38 ± 0.27	0.3	0.850
Systolic blood pressure response to standing, mmHg	1 ± 4	2 ± 7	3 ± 5	1 ± 9	3 ± 4	1.2	0.871
Diastolic blood pressure response to standing, mmHg	3 ± 3	7 ± 7	8 ± 8	7 ± 9	13 ± 4*	12.9	0.012
Heart rate response to standing, bpm	12 ± 6	11 ± 6	10 ± 6	17 ± 7	17 ± 9	9.2	0.057
Low-frequency heart rate variability spectral analysis	1.46 ± 0.57	1.75 ± 0.40	2.04 ± 0.90	2.04 ± 1.28	2.03 ± 1.03	0.8	0.536
High-frequency heart rate variability spectral analysis	2.64 ± 1.58	2.23 ± 0.92	2.77 ± 1.34	2.36 ± 1.44	2.48 ± 1.46	0.3	0.901

Data are means ± SD. Variables were compared among groups by Kruskall–Wallis’s *H*, univariate ANOVA, Welch-ANOVA, or univariate-GLM analysis adjusted by age if necessary, and followed by Tukey’s, Games-Howell’s, or Bonferroni’s *post hoc* analysis, as appropriate.

bpm, beats per minute; Ex-controls, healthy exercising control women with regular menses; Ex-FHA, women with exercise-induced functional hypothalamic amenorrhea; nonEx-controls, non-exercising control women with regular menses.

Pair-wise comparisons between women with Ex-FHA and other study subgroups.

* Participants showing statistically significant differences (*P *< 0.05) compared with women with Ex-FHA.

**Figure 2.**
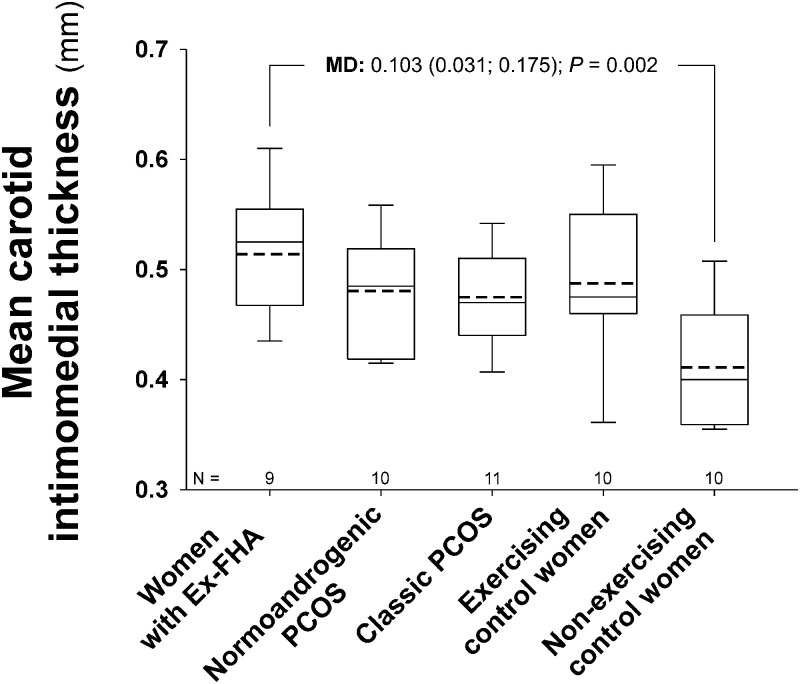
**Carotid intima-media thickness of women with Ex-FHA compared to individuals with PCOS and control participants**. The box indicates the 25th and 75th percentiles, the solid and short dashed lines within the boxes mark the median and mean, respectively. Whiskers below and above the box indicate the 10th and 90th percentiles. Mean carotid intima-media thicknesses were compared by univariate general linear models adjusting by age and BMI. Pair-wise mean differences between exercising women with Ex-FHA and other study subgroups were analyzed by the Bonferroni method. Ex-FHA, exercise-induced functional hypothalamic amenorrhea; MD, mean difference (95% CI) between women with Ex-FHA and nonEx-control women.

Regarding cIMT, women with Ex-FHA were the only subgroup of study participants showing significantly higher values than nonEx-controls, both on the right side [right cIMT: 0.520 ± 0.078 vs 0.408 ± 0.059 mm, respectively; MD: 0.112 (95% CI: 0.027; 0.197); *P *= 0.003] and on the left side [left cIMT: 0.508 ± 0.048 vs 0.414 ± 0.053 mm, respectively; MD: 0.094 (95% CI: 0.011; 0.177); *P *= 0.020]. Mean cIMT values showed similar findings (Fig. 2), even after adjusting by BMI.

**Figure 3.**
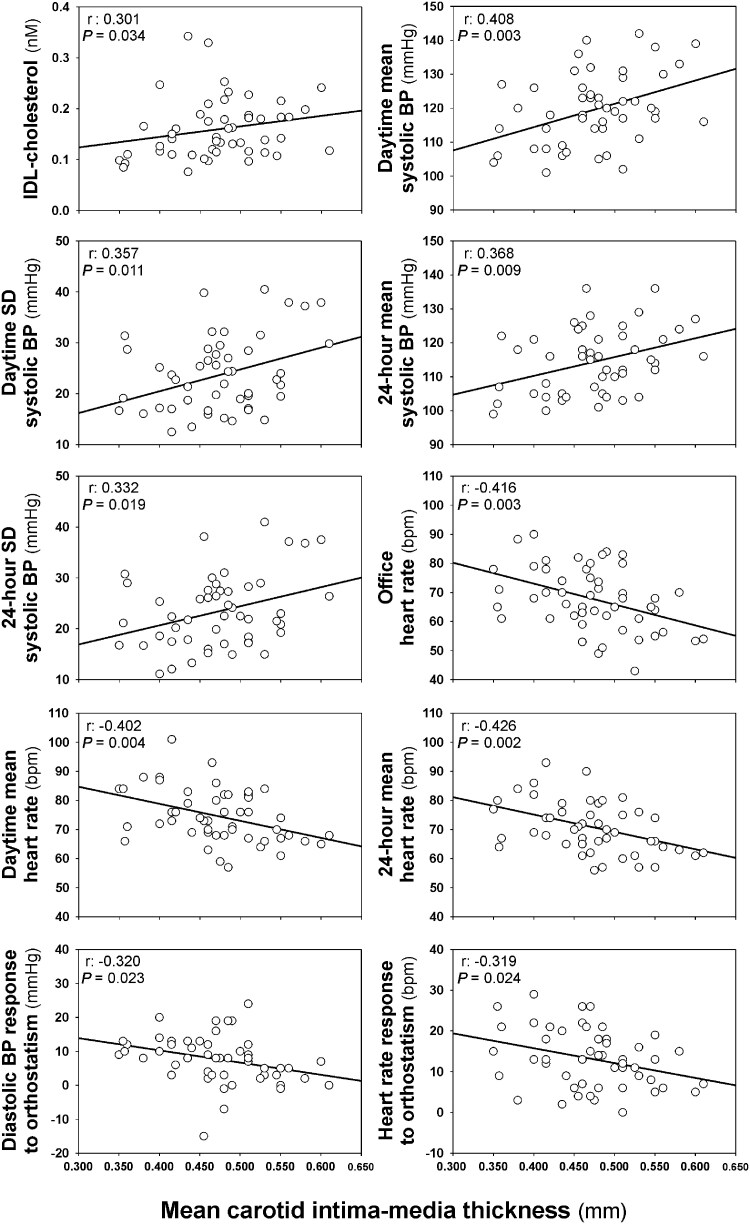
**Correlation analyses**. Panels describe statistically significant associations between carotid intima-media thickness and cardiometabolic parameters as assessed by Pearson’s or Spearman’s correlation analyses, considering all participants as a whole. BP, blood pressure; bpm, beats per minute; IDL, intermediate-density lipoprotein; *r*, Pearson’s coefficient of correlation; ρ, Spearman’s coefficient of correlation.

When we considered all participants as a whole, age, anthropometrics, BIE variables, and sex steroids showed no significant correlation with mean cIMT. Regarding carbohydrate and lipid parameters, mean cIMT was only correlated with IDL-cholesterol concentrations (Fig. 3). However, mean cIMT positively correlated with daytime mean systolic BP and its mean SD, and 24-h mean systolic BP and its mean SD. Supporting a pathogenic role of an increased vagal tone, mean cIMT was negatively associated with low HR recordings, such as office HR, daytime mean HR, 24-h mean HR, as well as with diastolic BP and HR responses to standing (Fig. 3).

## Discussion

We hereby report that adult women with Ex-FHA may show a greater cIMT than nonEx-control individuals, even after adjusting for BMI differences. Along with this finding, women with Ex-FHA showed subtle signs of autonomic dysfunction featured by parasympathetic overactivity. This condition included lower office HR and higher mean HR SD during daytime and 24-h periods than control women presenting with regular menses, independently of their exercising habits, or a lower diastolic BP response to standing than nonEx-control women. These women with Ex-FHA also showed higher HDL-cholesterol, HDL-triglycerides, large and medium HDL-P particles than both groups of women with PCOS and control women regardless of their physical activity.

Mean common cIMT is associated with future CV disease events, myocardial infarction, and stroke (Ling *et al.*, 2023). On the contrary, routine physical activity and less sedentary behavior are associated with less subclinical CV disease (Narendrula *et al.*, 2024). However, the presence of increased mean cIMT values in women with Ex-FHA with respect to healthy control women at very low-CV risk suggests that their long-term gonadal dysfunction might carry adverse consequences from a CV perspective, despite doing exercise on a regular basis.

In our series, cIMT was associated with parameters of cardiac autonomic dysfunction but not directly with hypoestrogenism when our study participants were considered as a whole. In line with our findings, low resting HR resulting from an augmented vagal modulation has been usually found in women with Ex-FHA (Tegg *et al.*, 2024). In consonance with this parasympathetic predominance, in the present study, participants with Ex-FHA exhibited a lower diastolic BP response to standing, as had been previously reported (O’Donnell *et al.*, 2015a). However, the negative association between HR and cIMT is not straightforward. A low HR variability down to sympathetic predominance is consistently associated with CV disease (Dekker *et al.*, 2000). Nevertheless, an increased vagal modulation as observed in women with Ex-FHA should induce an elevated HR variability (O’Donnell *et al.*, 2015b). Yet these women may also present with an augmented peripheral sympathetic vasoconstriction as a compensatory mechanism to maintain BP response to any orthostatic challenge (O’Donnell *et al.*, 2015a). Furthermore, the presence of hypoestrogenism may disrupt endothelial NO synthase-mediated peripheral vasodilation (Zahreddine *et al.*, 2021), contributing to peripheral vasoconstriction, and consequently, to potential carotid wall thickening.

Strikingly, women with Ex-FHA showed signs of subclinical atherosclerosis despite presenting with similar insulin sensitivity to control women, and apparently, a better lipid profile than the latter. In our study, cIMT was associated with well-known risk factors for subclinical atherosclerosis, such as circulating IDL-cholesterol concentrations (Liu *et al.*, 2023) or systolic BP when considering all participants as a whole. Nonetheless, a particular risk factor for subclinical atherosclerosis in women with Ex-FHA might be the existence of cholesterol-overloaded HDL particles. High levels of cholesterol-overloaded HDL particles have been independently associated with carotid atherosclerosis in asymptomatic individuals (Qi *et al.*, 2015), since excessive cholesterol enrichment of HDL particles impairs HDL reverse cholesterol transport capacity, and may turn HDL particles into cholesterol donors instead of acceptors. In line with this, extremely high levels of HDL-cholesterol are directly associated with CV disease in population-based studies (Madsen *et al.*, 2017) and in prospective cohort studies (Zhong *et al.*, 2020). Another plausible risk factor for subclinical atherosclerosis in women with Ex-FHA may be an impaired endothelial function, which is a consistent finding when these women are compared with eumenorrheic athletes and sedentary controls (Zeni Hoch *et al.*, 2003; Rickenlund *et al.*, 2005; Augustine *et al.*, 2016; Tegg *et al.*, 2024).

Incidentally, the hypoleptinemia resulting from low fat depots may further promote subclinical atherosclerosis in Ex-FHA. Food intake-regulating peptides also take part in CV control. Leptin concentrations are directly associated with greater cIMT in hypoestrogenic postmenopausal women (Everson-Rose *et al.*, 2021). However, leptin is suggested to be an important factor for vascular homeostasis and maintenance of vascular wall integrity in young healthy subjects (Schinzari *et al.*, 2013), by means of accelerating re-endothelialization and by decreasing neointima formation (Schroeter *et al.*, 2008). In any event, the present study did not examine circulating leptin levels.

In theory, these factors—i.e. cholesterol-overloaded HDL particles and/or hypoleptinemia—might confer an additional risk in women with Ex-FHA aside from a low endogenous estrogen exposure persisting in time during their reproductive years. Anyhow, all these mechanistic pathways are a mere hypothesis since the limited sample size of our study population precluded us from discriminating which were the main determinants of subclinical atherosclerosis in each single subgroup of women. For instance, hyperandrogenemia seems to be responsible for the presence of increased mean cIMT in women with classic PCOS compared with non-hyperandrogenic control women, besides insulin resistance and a high mean HR translating sympathetic overactivation (Luque-Ramírez *et al.*, 2007). Adiposity excess also becomes a main contributor to carotid subclinical atherosclerosis independently of PCOS phenotype (Pandurevic *et al.*, 2021). Despite Ex-FHA participants in our study showing similar cIMT values as those of women with classic and normoandrogenic PCOS, none of those factors played a role in the subclinical atherosclerosis of the former. Therefore, larger confirmatory studies are needed to establish cIMT as an early marker of atherosclerosis in Ex-FHA and to clarify which intrinsic risk factors underlie this association.

Other weaknesses in our study were: (i) its cross-sectional and observational design, which does not allow causal inference regarding associations between independent variables, such as autonomic dysfunction, and cIMT; and (ii) multiplicity, which may lead to spurious associations in some cases (type 1 error) despite having implemented rigorous adjustments for multiple comparisons whenever possible.

In spite of these shortcomings, our study also had several strengths. We highlight the careful phenotyping of patients and controls using state-of-the-art methodology for sex steroid measurements, the robustness of proton nuclear magnetic resonance spectroscopy for lipid assessment, and the comprehensive evaluation of BP recordings and cardioautonomic function.

In conclusion, our novel finding regarding subclinical carotid atherosclerosis may support prior scientific literature suggestive of functional amenorrhea disrupting CV physiology (Tegg *et al.*, 2024) and potentially exposing women with Ex-FHA to CV morbidity in their future. Women with Ex-FHA show signs of a parasympathetic tone upregulation beyond physical training, which appears to be associated with higher cIMT values. As expected, the cardiometabolic Ex-FHA phenotype includes very low adiposity and high insulin sensitivity indices; but a theoretically beneficial lipid profile consisting of HDL-cholesterol and HDL particles might turn, speculatively, into deleterious.

Robust data on CV endpoints in the long term of women with Ex-FHA are lacking. Thus, larger studies are warranted to draw definitive conclusions about the role of carotid measurements in the CV phenotyping of exercising women with gonadal dysfunction due to a negative energy balance.

## Data Availability

All datasets generated and/or analyzed during the current study are not publicly available but are available from the corresponding author on reasonable request.
